# A pilot study of the impact of massage therapy on symptom burden during infusion in patients with cancer

**DOI:** 10.3389/fonc.2026.1943581

**Published:** 2026-10-19

**Authors:** Christy DiCristofano, Marianne Razavi, Phi Hoang Tran, Sarah Ku, Aaron Fisher, Elyssa Kim, Judith Rose, Nicol Hedgpeth, Jamie Fertal, Richard T. Lee

**Affiliations:** 1 Cherng Family Center for Integrative Oncology, City of Hope National Medical Center, Duarte, CA, United States; 2 Department of Supportive Care Medicine, City of Hope National Medical Center, Duarte, CA, United States; 3 Nursing Administration, City of Hope National Medical Center, Duarte, CA, United States; 4 Department of Medical Oncology, City of Hope National Medical Center, Duarte, CA, United States

**Keywords:** chemotherapy infusion, massage, oncology, quality of life, symptoms

## Abstract

**Background:**

Massage therapy (MT) is a popular integrative therapy sought by patients with cancer that improves quality of life. This pilot study assesses the therapeutic effects of massage therapy on patient reported symptoms during chemotherapy infusion.

**Methods:**

This retrospective study evaluated outcomes from patients receiving a hand and/or foot massage from 2023–2024 during chemotherapy infusion at a comprehensive cancer center. Patients completed a pre- and post-massage modified Edmonton Symptom Assessment Scale (ESAS) using a numerical scale from 0–10. A questionnaire was provided to infusion nurses to assess their perspective. Mixed effects linear regression models were used to evaluate changes in symptoms incorporating massage type, its interaction with time, and demographic covariates.

**Results:**

A total of 313 patients received 652 MT sessions and included for analyses. The participants were mostly female (74%) with a median age of 62. The most common types of cancer in our patient population were hematologic (28%), gastrointestinal (20%), and breast (19%). The number of massage sessions ranged from: 1 (48%) 2 (17%), 3 (9%), and ≥4 (26%). Patients equally chose foot and hand massage (13% each) while most chose combined hand and foot massage (74%). At baseline, the most severe reported symptoms were tiredness (mean=3.8 ± 2.7 SD), neuropathy (3.2 ± 3.3), wellbeing concerns (3.2 ± 2.6), and anxiety (2.3 ± 2.6). Mixed effect models showed pre-/post- symptom burden reductions in all symptoms: nausea, pain, tiredness, anxiety, depression, shortness of breath, neuropathy and wellbeing concerns (Time effect F-statistic ranging from 26-129, p-values < 0.001). The largest improvements were seen with neuropathy (1.5, ± 0.13 SE), anxiety (1.2, ± 0.10), wellbeing (1.2, ± 0.13), and tiredness (1.2, ± 0.13). There were no between-massage group differences in pre–post change for any symptom (time × massage not significant). However, the magnitude of within-massage group improvement varied across modalities, with combination, and foot only massages, showing greater improvement than hand massage.

**Conclusion:**

Based on this pilot study, MT is a feasible adjunctive treatment for patients receiving chemotherapy infusion, with no reported complications. Massage was associated with reducing symptoms such as neuropathy, anxiety, and tiredness. Further research is needed to evaluate the benefits of MT during infusion.

## Introduction

Massage therapy for patients with cancer is a developing field that addresses the profound impact of a cancer diagnosis and its treatment. Patients often seek non-pharmacological treatments to address symptoms and improve quality of life, and in this context massage therapy emerges as a powerful and supportive intervention for individuals undergoing cancer treatment.

Research has consistently shown that massage therapy provides multiple benefits for cancer patients, including reductions in pain, stress, and anxiety, as well as improvements in mood and overall well-being. These effects have been supported by both observational studies and randomized controlled trials (1–3). The Society for Integrative Oncology (SIO) and the American Society of Clinical Oncology (ASCO) have provided guidelines on integrative oncology care, recommending massage therapy as a supportive intervention for managing symptoms of pain in adults with cancer (4).

Despite growing evidence supporting massage therapy’s benefits, there remains a significant gap in understanding its role when administered specifically during anti-cancer infusion treatments. Limited research has explored how massage during infusion affects anxiety, pain levels, and other symptoms, leaving a crucial area underexamined. This gap emphasizes the need for targeted studies to better understand and optimize the timing and context of massage therapy in oncology care. This pilot study assesses the effects of massage therapy on patients’ symptoms while receiving anti-cancer treatment infusions. We further administrated a survey to nurses that assessed their perception of massage importance and feasibility.

## Methods

A pilot program was conducted over 12 months during 2023–2024 at the City of Hope Lennar Foundation Cancer Center’s infusion center as a clinical service initiative aimed at providing complimentary hand and/or foot massage therapy on patients undergoing chemotherapy infusion. The objective was to assess whether massage therapy could be provided without interrupting clinical care and assess the impact on symptoms and quality of life during chemotherapy infusion. This study represents a retrospective evaluation of a pilot quality improvement project, in which data collected during routine clinical care were analyzed retrospectively. This project was reviewed by the City of Hope Institutional Review Board and was determined to be a retrospective quality improvement initiative not constituting human subjects research (IRB #24191). Eligible patients receiving chemotherapy were offered the option to participate in the program during their scheduled infusion appointments. Infusion nurses introduced the initiative and provided a brief explanation of the potential benefits of massage therapy.

Patients who opted in, provided written consent through a standardized “Consent to Treat” form. This consent process was part of standard of care for any types of procedures at City of Hope and was not conducted for research purposes. Patients were asked whether they would like to receive a hand and/or foot massage during their treatment. A licensed massage therapist then performed a 15–20-minute effleurage massage with lotion (Biotone Massage Cream), targeting either the hands, feet, or both, depending on the patient’s preference. Effleurage techniques involve long, gentle, gliding strokes designed to promote relaxation and circulation without applying deep pressure (1–3 on Walton pressure scale), making them suitable for medically fragile patients.

Immediately before receiving the massage, each participant completed a pre-intervention modified Edmonton Symptom Assessment System (ESAS), utilizing a 0–10 numerical rating scale to assess symptoms such as pain, anxiety, tiredness, nausea, depression, shortness of breath, neuropathy, constipation, and overall well-being (**Supplemental Material 2**). Directly following the session, patients completed a post-intervention survey using the same modified ESAS scale, enabling pre- and post-treatment comparisons (**Supplemental Material 3**). These assessments were collected by either massage therapist or nurse as part of routine clinical practice and later analyzed retrospectively. In addition to symptom ratings, demographic information, cancer staging, type of chemotherapy regimen, and the number of massage sessions received per patient were recorded for subsequent analysis. The infusion nursing staff completed a questionnaire assessing changes in patient and nurse stress levels, symptom burden, the impact of the massage service on nursing tasks, and the perceived importance of offering massage during infusion.

Descriptive summaries were produced. Mixed effects linear regression models were used to evaluate time (pre to post massage changes) in symptom scores, accounting for repeated measurements within individuals. Each symptom (nausea, pain, tiredness, anxiety, depression, shortness of breath, neuropathy, and wellbeing) was modeled separately. All models included fixed effects for time (pre vs. post), massage type (hand, foot, or both), and time × massage type interaction (to assess whether pre–post changes differed by massage modality); age and gender were included as covariates. Models were fitted using restricted maximum likelihood with the Kenward–Roger method. Overall effects of massage type were evaluated using Type III F tests; slice tests were used to examine within group pre-post change for each massage type; Tukey adjusted pairwise comparisons were applied to all relevant categorical contrasts, including differences between massage modalities. All available data was used. Analyses were conducted using SAS (proc mixed, which accommodates missingness in repeated measures data). Statistical significance was considered for p<0.05.

## Results

In this pilot program, 313 patients received massage therapy during anti-cancer treatment infusion. Most were female (74%), median age of 62 years old, and self-identified as being White (57%), Asian (19%), and Hispanic/Latino (11%). The most common types of cancer were hematologic (28%), gastrointestinal (20%), breast (19%), and gynecologic (16%) with a range of cancer stages: I 11%, II 13%, III 15%, IV 31%\. (see **Table 1**). A total of 652 hand and/or foot massage sessions were provided with participants receiving a median of 2 sessions (1–24 session). The majority selected both a combined hand and foot massage (74%) while others selected hand or foot massage equally (13% each). At baseline, the most severe symptoms (Mean, SD) reported were tiredness (3.8 ± 2.7), neuropathy (3.2 ± 3.3), wellbeing concerns (3.2 ± 2.6), and anxiety (2.3 ± 2.6).

**Table 1:** N = 313.

Characteristic	Category	Count	Frequency
Gender			
	Female	230	73.5%
	Male	78	24.9%
	Blank/Decline to Answer	5	1.6%
Race/ethnicity			
	White	177	56.5%
	Asian	58	18.5%
	Hispanic or Latino	33	10.5%
	Other	20	6.4%
	Decline to Answer	19	6.1%
	Black or African American	6	1.6%
Cancer type			
	Hematologic	86	27.5%
	Gastrointestinal	61	19.5%
	Breast	60	19.2%
	Gynecologic	51	16.3%
	Other	55	17.6%
Cancer stage			
	I	35	11.2%
	II	42	13.4%
	III	47	15.0%
	*IV*	96	30.7%
	*Unknown*	93	29.7%

Pre- and post-session symptom scores, measured by ESAS, revealed statistically significant improvements across all evaluated domains. (**Figure 1**, **Table 2**), with mixed-effects models confirming overall improvements in neuropathy (1.46, SE = 0.13), tiredness (1.21, SE = 0.13), wellbeing (1.20, SE = 0.13), anxiety (1.19, SE = 0.12), pain (0.87, SE = 0.12), depression (0.59, SE = 0.10), nausea (0.50 SE = 0.10), and shortness of breath (0.45, SE = 0.09). Time-effect F-statistics were ranging from 26 to 128, all p-values < 0.001 (**Table 3B**). Thus, improvements of at least one point were observed for several symptoms, with the largest reductions seen for neuropathy, tiredness, wellbeing, and anxiety. The time × massage type interaction was not significant for any outcome, indicating that overall, pre-post changes did not differ across massage modalities (**Table 3B**). However, slice tests revealed symptom-specific patterns of response: certain outcomes improved in some massage groups but not others, and the estimated magnitude of within-massage group improvement varied across modalities, with combination massage, and foot massage, showing larger estimated improvements than hand (**Table 3A**). These *post hoc* modality-specific estimates are presented descriptively.

**Figure 1:**
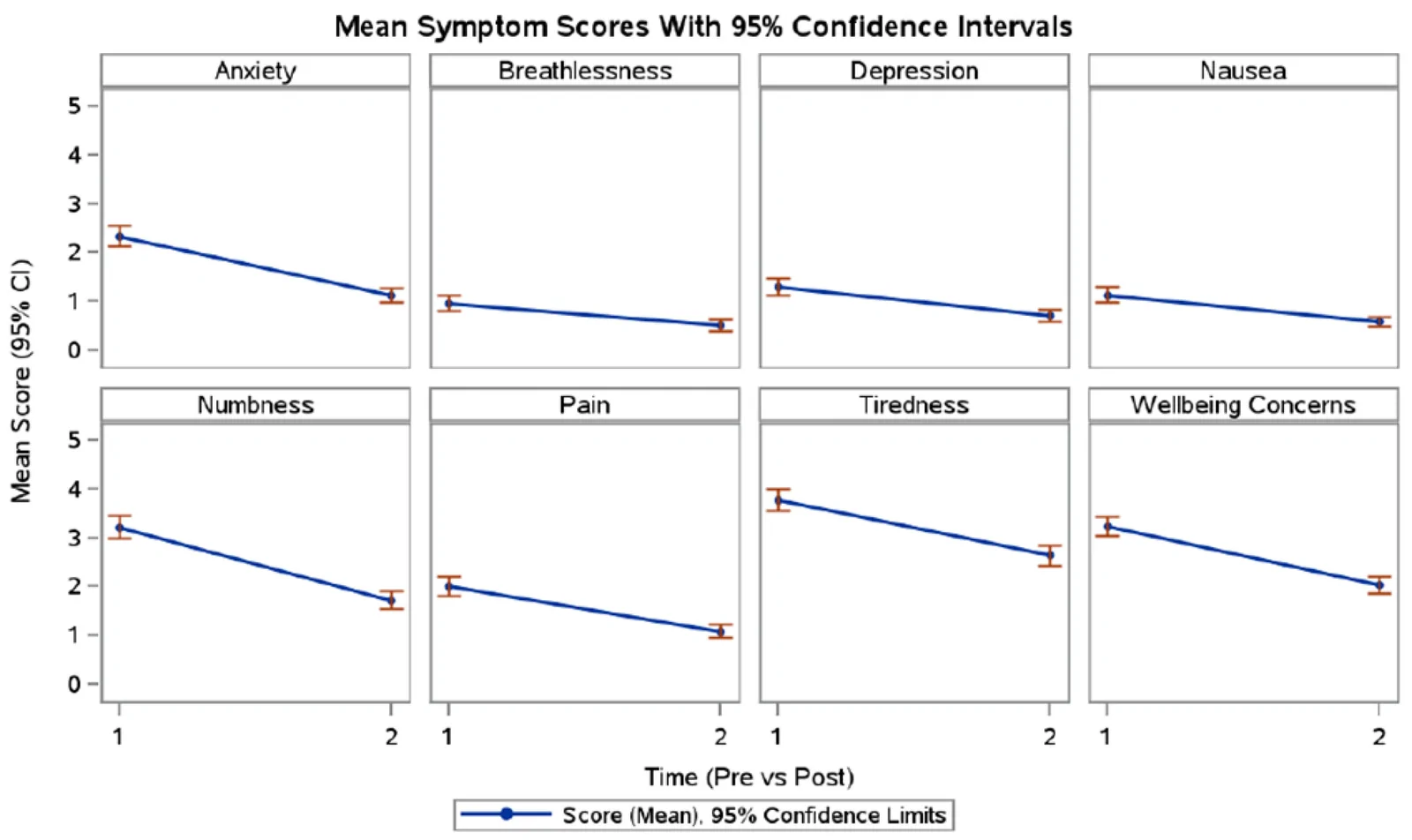
Pre/post mean symptom scores (updated for 8 symptoms instead of 10).

**Table 2:** Symptoms scores at baseline and pre-post change after massage (from mixed effects models).

Symptom	Symptom frequency (%)	Baseline mean score (± SD)	Mean change (± SE)
Anxiety	59.3%	2.34 (2.64)	1.19 (0.12)
Shortness of breath	25.9%	0.97 (2.02)	0.45 (0.09)
Depression	36.2%	1.30 (2.17)	0.59 (0.10)
Nausea	33.9%	1.14 (1.99)	0.50 (0.10)
Neuropathy	65.2%	3.22 (3.34)	1.46 (0.13)
Pain	51.3%	2.01 (2.52)	0.87 (0.12)
Tiredness	82.8%	3.78 (2.74)	1.21(0.13)
Wellbeing	77.9%	3.24 (2.57)	1.20 (0.13)

**Table 3A:** Estimated within-group pre-post symptom changes by massage type (slice tests).

Symptom	Hand	Foot	Combination
	change (p-value)	change (p-value)	change (p-value)
Nausea	Not significant	0.51 (p = 0.012)	0.60 (p < 0.0001)
Pain	0.49 (p = 0.041)	1.15 (p < 0.001)	0.98 (p < 0.0001)
Tiredness	1.06 (p < 0.001)	1.46 (p < 0.001)	1.11 (p < 0.001)
Anxiety	1.00 (p < 0.001)	1.35 (p < 0.001)	1.23 (p < 0.001)
Depression	Not significant	0.80 (p < 0.001)	0.59 (p < 0.001)
Shortness of Breath	Not significant	0.60 (p < 0.001)	0.45 (p < 0.001)
Neuropathy/Numbness	1.17 (p < 0.001)	1.65 (p < 0.001)	1.57 (p < 0.001)
Wellbeing	0.90 (p < 0.001)	1.46 (p < 0.001)	1.23 (p < 0.001)

Values represent estimated pre-post symptom improvement within each massage modality obtained from slice tests.

Nausea decreased significantly in combination (0.60, p <.0001), and foot (0.51, p= 0.0116), but not in hand. Pain decreased across all modalities: combination (0.98, p <.0001), foot (1.15, p<0.001) and hand (0.49, p=0.041); as did tiredness: combination (1.11, p < 0.001), foot (1.46, p < 0.001), and hand (1.06, p < 0.001). Anxiety also improved across all groups: combination (1.23, p < 0.001), foot (1.35, p <0.001), and hand (1.00, p < 0.001). Depression only decreased in combination (0.59, p < 0.001) and foot (0.80, p < 0.001). Shortness of breath improved only in combination (0.45, p < 0.001) and foot (0.60, p< 0.001). Neuropathy decreased across all massage modalities: combination (1.57, p< 0.001), foot (1.65, p<0.001), and hand (1.17, p< 0.001); as did wellbeing: combination (1.23, p<0.001), foot (1.46, p<0.001), and hand (0.90, p<0.001) (**Table 3A**).

Furthermore, analyses showed that patients selecting different massage types differed in symptom burden. Indeed, a significant main effect of massage type was observed for pain (F = 9.01, p<0.001), tiredness (F = 3.98, p=0.0190), and neuropathy (F = 3.52, p=0.0298) (**Table 3B**). For these symptoms, patients selecting hand massage reported lower symptom scores than those choosing combination or foot massage. Tukey-adjusted comparisons revealed that, relative to patients who choose hand-massage, combination was associated with higher pain (0.91, p=0.0001), tiredness (0.65, p=0.0354) and neuropathy (0.67, p=0.025), while foot-massage was associated with higher pain (0.94, p=0.0015) and tiredness (0.85, p=0.0216). Older age was related to slightly lower nausea level (0.014, p=0.0177), but greater shortness of breath (0.021, p=0.0013).

**Table 3B:** Adjusted mixed-effects models across individual symptoms (F-statistics, p-values).

Effect	Nausea	Pain	Tiredness	Anxiety	Depression	Shortness of breath	Neuropathy	Wellbeing
Time	25.73 (**<.001**)	54.84 (**<.001**)	82.64 (**<.001**)	102.19 (**<.001**)	37.75 (**<.001**)	27.77 (**<.001**)	128.84 (**<.001**)	83.40 (**<.001**)
Age	5.69 (.**0177**)	0.68 (.4099)	0.24 (.6267)	3.68 (.0559)	0.24 (.6256)	10.54 (.**0013**)	2.70 (.1011)	0.04 (.8471)
Gender	0.20 (.6529)	0.05 (.8217)	3.27 (.0713)	1.34 (.2474)	0.14 (.7066)	0.13 (.7215)	0.36 (.5466)	0.25 (.6145)
Massage Type	0.30 (.7407)	9.01 (**.0001**)	3.98 (**.0190**)	2.66 (.0705)	2.38 (.0932)	1.18 (.3064)	3.52 (**.0298**)	1.07 (.3432)
Time × Massage Type	0.58 (.5606)	2.25 (.1062)	0.75 (.4730)	0.57 (.5635)	1.23 (.2925)	0.74 (.4791)	1.15 (.3157)	1.15 (.3159)

Mixed-effects regression models estimated symptom scores before and after massage while adjusting for age and sex. The Time effect tests whether symptoms improved following massage. The Massage Type effect tests whether symptom scores differed among patients selecting hand, foot, or combination massage. The Time × Massage Type effect tests whether patterns of symptom improvement varied across massage modalities. Age was associated with nausea and shortness of breath; no significant sex effects were observed. Bolded values indicated statistical significance.

No adverse events or complications related to massage therapy were reported over the course of the program. Patient engagement remained high, and feedback was overwhelmingly positive, reinforcing the acceptability of the intervention in this setting. (See **Supplemental Material 1**).

### Nurse survey results

Toward the end of the pilot program, a survey was distributed to twenty-eight infusion nurses (**Supplemental Material 4**). Twenty-two complete surveys were received from respondents identified as chemotherapy infusion nurses who have cared for patients receiving massage therapy during infusion. Nurses rated the impact of massage therapy on patients’ stress levels highly, 4.9 out of 5. The average rating for improvement in patients’ overall symptom burden was 4.4. Nurses also reported a positive effect on their own stress or burden, with a mean score of 4.4. Importantly, massage therapy was not perceived to disrupt nursing tasks, receiving a unanimous score of 1 (no disruption) from all respondents. The perceived importance of massage therapy for patients in the infusion area was rated 4.9. Preferences for timing of massage therapy varied, with most nurses favoring therapy during chemotherapy, at any time, and during pre-medications (**Figure 2**).

**Figure 2:**
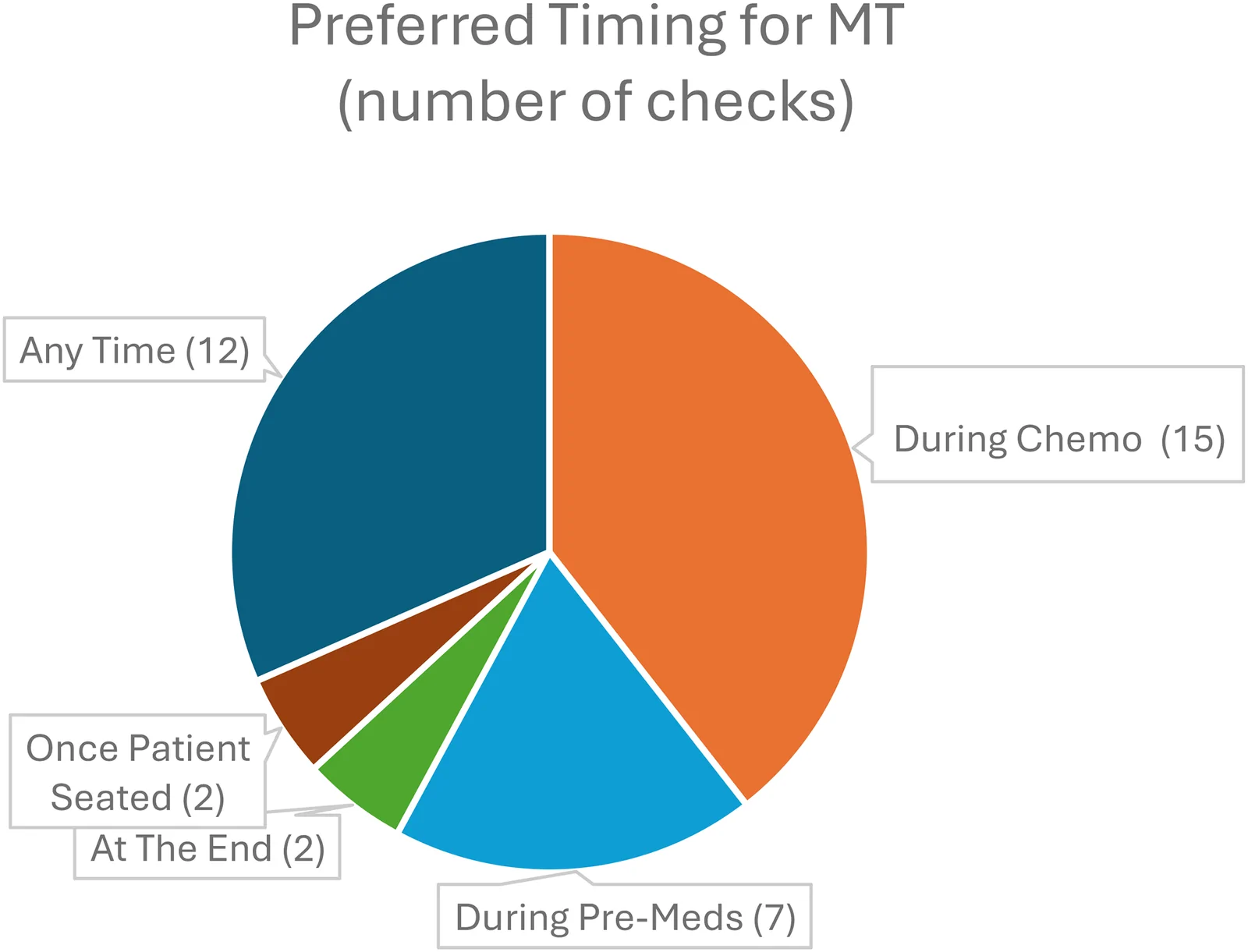
Pie chart representing nurse survey responses for preferred timing of massage therapy (n=38).

## Discussion

This pilot study represents one of the largest reports of massage therapy during chemotherapy infusion and associated changes. This data demonstrates that implementing massage therapy during chemotherapy infusion is both feasible and clinically beneficial. Patients reported clinically significant improvements (>1/10) in a range of physical and psychological symptoms, especially with neuropathy, tiredness, wellbeing, and anxiety—symptoms that are frequently reported as distressing during cancer treatment. Importantly, the intervention was well received by both patients and nursing staff The absence of reported complications suggests that effleurage massage, administered by trained therapists, is a safe adjunctive therapy in the oncology setting. Our findings align with the broader evidence supporting massage therapy in oncology settings and are consistent with current clinical guidelines.

Our results are similar to other published studies examining the benefits of massage for patients with cancer. An observational study of 1,290 cancer patients over 3 years found that massage therapy reduced symptom scores between 21-54% (2). One of the largest randomized control trials of massage therapy for patients with advanced cancer by Kutner et al. found significant improvements in both pain (1.87 points out of 10) and mood (1.58) as compared to a simple touch control (1). Most recently, IMPACT (Integrative Medicine for Pain in Patients with Advanced Cancer Trial), a multicenter pragmatic randomized controlled trial led by Mao et al. enrolled 298 patients with advanced cancer and moderate-to-severe musculoskeletal pain. Patients received weekly massage or acupuncture for 10 weeks followed by monthly booster sessions up to 26 weeks. Massage therapy reduced worst pain scores by a mean of 3.01 points (95% CI -3.38 to -2.63) and improved tiredness, insomnia, and quality of life, with no significant difference between massage and acupuncture. The authors concluded that these nonpharmacologic interventions can improve pain and symptom control while potentially reducing medication use (5).

The improvement in neuropathy symptoms is particularly noteworthy, as peripheral neuropathy is a common and often treatment-limiting side effect of many chemotherapeutic agents. A randomized pilot study of 71 patients by Lopez et al. examined oncology massage for chemotherapy-induced peripheral neuropathy (CIPN), comparing different massage schedules. Patients who received the more intensive regimen—three sessions weekly for four weeks—showed greater improvements in self-reported CIPN pain and sensory symptoms than those in the less intensive schedule (3). In this study, the Pain Quality Assessment Scale scores showed an improvement of 1.19 to 1.68 (p<0.05) on the three subscales of deep pain, paroxysmal pain, and surface pain. A recent retrospective study similarly reported significant neuropathy reduction (-1.2 points) following a single massage session (6). Whether massage therapy is a strategy to consider while receiving chemotherapy associated with CIPN is unclear and requires further evaluation. Additionally, the mechanism through which massage reduces neuropathy symptoms remains to be fully understood, increased circulation, relaxation, and sensory modulation may play contributory roles.

A systematic review by Calcagni et al. highlighted that manipulative and body-based practices, including massage, show positive effects on symptom outcomes, particularly pain and tiredness, although the quality of evidence is often limited by methodological issues (7). Additionally, a meta-analysis by Win Myint et al. found that massage therapy significantly improves cancer pain, quality of life, and anxiety levels, despite the very low certainty of evidence (8). Another meta-analysis by Shan et al. demonstrated that massage therapy significantly improves cancer-related tiredness, particularly in breast cancer patients. Reflexology and Chinese massage were found to be more effective approaches, with optimal intervention frequency being twice a week for 3–5 weeks and a duration of 20–40 minutes per session (9). Consistent with these reviews, the SIO-ASCO guidelines recommend massage therapy for cancer-related pain, noting intermediate-quality evidence and moderate strength of recommendation supporting its use as an adjunctive treatment for cancer pain (4).

Beyond symptom relief, our study provides important implementation data: nursing staff rated massage therapy highly beneficial for patient stress (4.9/5) and symptom burden (4.4/5) while reporting no disruption to clinical workflow, supporting the feasibility of integrating massage into the infusion setting. The consistency of improvements across multiple domains and high acceptability among patients and staff suggest massage therapy during chemotherapy infusion is a promising supportive care intervention.

This study also provides a potential solution to a major barrier to receiving massage therapy- cost (10). By providing massage therapy during treatment infusions, this should lower the cost of delivery because the therapy is provided at a standard point of care already being delivered. Thus, the room and support staff are already provided as opposed to scheduling the patient for a separate massage appointment in a different clinic and time. In this service delivery model, the only additional cost is the massage therapist. Such a delivery model is also beneficial for the patient as no added burden is created to receive massage therapy such as an additional trip for another appointment. Addressing financial and operational barriers including insurance reimbursement is essential to ensure equitable access and sustainability across diverse healthcare settings.

### Limitations

This study has several limitations. The results are from a pilot program providing prospective observational data about the changes associated with massage therapy during chemotherapy infusion. The lack of a control group prevents clear attribution of improvements to the massage intervention, as other factors may have influenced outcomes. While pre- and post-session assessments captured immediate symptom changes, they did not assess the duration or long-term effects of treatment. Additionally, within our study both the massage therapist and nurses collected post-surveys. Having the massage therapist collect surveys may introduce bias from patients who want to support the massage therapist with positive outcomes. Conclusions from this study have limited generalizability because it was conducted at a single center with one massage therapist.

## Conclusion

This pilot study supports the use of massage therapy as a feasible adjunctive treatment for managing symptoms in patients undergoing anticancer treatment infusions. The intervention was associated with consistent improvements in key physical and emotional symptoms, including neuropathy, anxiety, and tiredness. Importantly, no complications or adverse effects were reported, highlighting the safety and tolerability of this non-invasive supportive therapy within a clinical infusion setting. Additionally, a survey of nurses attending to patients within this study reported massage therapy lowering patients’ stress levels and improving patient symptom burden. After patient massage therapy, nurses also reported a positive effect on their own stress and burden and perceived the therapy as nondisruptive to nursing tasks. The integration of massage therapy into routine cancer care has the potential to enhance the overall treatment experience, promote patient comfort, and contribute to improved quality of life. However, broader implementation requires further investigation including examining long-term effects.

Future research should prioritize randomized controlled trials especially to optimize dosing parameters (frequency, duration, technique), inclusion of diverse patient populations, and comprehensive evaluations of clinical efficacy (including longer follow-up), cost-effectiveness, impact on healthcare utilization (e.g., reducing medication uses or emergency visits), and patient, family, and healthcare provider perspectives, which should support insurance reimbursement. Anecdotally, we found that massage reduced patient blood pressure, allowing several patients to proceed with chemo infusions that had previously been delayed due to hypertension. Future studies may investigate this further, exploring outputs such as patient blood pressure and chemotherapy delay times. As the field of integrative oncology continues to grow, evidence-based interventions like massage therapy may play a vital role in the holistic care of patients with cancer.

## Data Availability

The raw data supporting the conclusions of this article will be made available by the authors, upon reasonable request, without undue reservation.

## Supplementary material

Supplementary Material 1 Patient feedback on massage therapy during chemotherapy: Patients consistently described having an “emotional release” such as “crying” and feeling “better after.” Massage therapy helped reduce anxiety and stress, promoting relaxation and improved mood. Physically, patients noted pain relief in the back, abdomen, and extremities, increased flexibility, and lasting neuropathy symptom improvement. Several patients experienced lowered blood pressure after massage, allowing them to proceed with chemotherapy that had been delayed due to hypertension. Appreciation for massage was high, with some calling it “the best massage” and expressing strong desire for the service to continue. Many even adjusted their treatment schedules to coincide with massage availability, highlighting its value in their care. Nurse feedback on patient experience with massage therapy: “Every patient who receives massage therapy asks for it again,” reflected one nurse, capturing the overwhelmingly favorable patient feedback reported. Nurses consistently observed that patients felt calmer, more relaxed, and expressed deep appreciation for massage therapy during infusion. Many noted that patients frequently requested massages at subsequent visits, highlighting its emotional and therapeutic value as “human touch” that eases anxiety and stress. Nurses reported improvements in peripheral neuropathy, reduced blood pressure allowing treatment to proceed, and enhanced overall well-being. Massage therapy was described as an essential component of care, with patients often scheduling treatments around its availability. The service was credited with setting the infusion experience apart from other centers, improving patient satisfaction and comfort throughout chemotherapy.
