# Supplementary material for: A pilot study of the impact of massage therapy on symptom burden during infusion in patients with cancer
Source: Front Oncol. 2026 Oct 19;16:1943581. doi: 10.3389/fonc.2026.1943581 (PMC13724564; doi:10.3389/fonc.2026.1943581)
Supplement: Supplementary file 1 [file Supplementaryfile1.docx]

**Supplemental Material 1**

**Patient feedback on massage therapy during chemotherapy:**

Patients consistently described having an “emotional release” such as “crying” and feeling “better after.” Massage therapy helped reduce anxiety and stress, promoting relaxation and improved mood. Physically, patients noted pain relief in the back, abdomen, and extremities, increased flexibility, and lasting neuropathy symptom improvement. Several patients experienced lowered blood pressure after massage, allowing them to proceed with chemotherapy that had been delayed due to hypertension. Appreciation for massage was high, with some calling it “the best massage” and expressing strong desire for the service to continue. Many even adjusted their treatment schedules to coincide with massage availability, highlighting its value in their care.

**Nurse feedback on patient experience with massage therapy:**

“Every patient who receives massage therapy asks for it again,” reflected one nurse, capturing the overwhelmingly favorable patient feedback reported. Nurses consistently observed that patients felt calmer, more relaxed, and expressed deep appreciation for massage therapy during infusion. Many noted that patients frequently requested massages at subsequent visits, highlighting its emotional and therapeutic value as “human touch” that eases anxiety and stress. Nurses reported improvements in peripheral neuropathy, reduced blood pressure allowing treatment to proceed, and enhanced overall well-being. Massage therapy was described as an essential component of care, with patients often scheduling treatments around its availability. The service was credited with setting the infusion experience apart from other centers, improving patient satisfaction and comfort throughout chemotherapy.

**Supplemental Material 2**

**Massage During Infusion Pre-Survey**

Patient Name: _____________________________MRN: __________________DOB: _______________

Type of cancer: ________________________

Please fill out the survey to the best of your ability. Thank you.

| Nausea (sick feeling in stomach, vomiting) | | | | | | | | | | | | |
| --- | --- | --- | --- | --- | --- | --- | --- | --- | --- | --- | --- | --- |
|  | | | | | | | | | | | | |
| None | 0 | 1 | 2 | 3 | 4 | 5 | 6 | 7 | 8 | 9 | 10 | Worst  Possible |

Mild	Moderate	Severe	Very Severe

| Pain | | | | | | | | | | | | |
| --- | --- | --- | --- | --- | --- | --- | --- | --- | --- | --- | --- | --- |
|  | | | | | | | | | | | | |
| None | 0 | 1 | 2 | 3 | 4 | 5 | 6 | 7 | 8 | 9 | 10 | Worst  Possible |

Mild	Moderate	Severe	Very Severe

| Tiredness (less energy, fatigue) | | | | | | | | | | | | |
| --- | --- | --- | --- | --- | --- | --- | --- | --- | --- | --- | --- | --- |
|  | | | | | | | | | | | | |
| None | 0 | 1 | 2 | 3 | 4 | 5 | 6 | 7 | 8 | 9 | 10 | Worst  Possible |

Mild	Moderate	Severe	Very Severe

| Anxiety (distress, worry, or being nervous) | | | | | | | | | | | | |
| --- | --- | --- | --- | --- | --- | --- | --- | --- | --- | --- | --- | --- |
|  | | | | | | | | | | | | |
| None | 0 | 1 | 2 | 3 | 4 | 5 | 6 | 7 | 8 | 9 | 10 | Worst  Possible |

Mild	Moderate	Severe	Very Severe

| Depression (feeling sad or blue) | | | | | | | | | | | | |
| --- | --- | --- | --- | --- | --- | --- | --- | --- | --- | --- | --- | --- |
|  | | | | | | | | | | | | |
| None | 0 | 1 | 2 | 3 | 4 | 5 | 6 | 7 | 8 | 9 | 10 | Worst  Possible |

Mild	Moderate	Severe	Very Severe

| Shortness of breath | | | | | | | | | | | | |
| --- | --- | --- | --- | --- | --- | --- | --- | --- | --- | --- | --- | --- |
|  | | | | | | | | | | | | |
| None | 0 | 1 | 2 | 3 | 4 | 5 | 6 | 7 | 8 | 9 | 10 | Worst  Possible |

Mild	Moderate	Severe	Very Severe

Neuropathy (numbness, tingling, and/or burning sensation)

| None | 0 | 1 | 2 | 3 | 4 | 5 | 6 | 7 | 8 | 9 | 10 | Worst  Possible |
| --- | --- | --- | --- | --- | --- | --- | --- | --- | --- | --- | --- | --- |
| Mild Moderate Severe Very Severe  Overall Well-being | | | | | | | | | | | | |
|  | | | | | | | | | | | | |
| Best | 0 | 1 | 2 | 3 | 4 | 5 | 6 | 7 | 8 | 9 | 10 | Worst  Possible |

Mild	Moderate	Severe	Very Severe

To be completed by massage therapist.

1. What kind of massage was done on the patient?

- Hand
- Foot
- Both

2. Where was the patient for the massage session?

- Infusion

**Supplemental Material 3**

**Massage During Infusion Post Survey**

Patient Name: _____________________ MRN:___________DOB:__________

| Nausea (sick feeling stomach, vomiting) | | | | | | | | | | | | |
| --- | --- | --- | --- | --- | --- | --- | --- | --- | --- | --- | --- | --- |
|  | | | | | | | | | | | | |
| None | 0 | 1 | 2 | 3 | 4 | 5 | 6 | 7 | 8 | 9 | 10 | Worst  Possible |

Mild	Moderate	Severe	Very Severe

| Pain | | | | | | | | | | | | |
| --- | --- | --- | --- | --- | --- | --- | --- | --- | --- | --- | --- | --- |
|  | | | | | | | | | | | | |
| None | 0 | 1 | 2 | 3 | 4 | 5 | 6 | 7 | 8 | 9 | 10 | Worst  Possible |

Mild	Moderate	Severe	Very Severe

| Tiredness (less energy and fatigue) | | | | | | | | | | | | |
| --- | --- | --- | --- | --- | --- | --- | --- | --- | --- | --- | --- | --- |
|  | | | | | | | | | | | | |
| None | 0 | 1 | 2 | 3 | 4 | 5 | 6 | 7 | 8 | 9 | 10 | Worst  Possible |

Mild	Moderate	Severe	Very Severe

| Anxiety (distress, worry, or being nervous) | | | | | | | | | | | | |
| --- | --- | --- | --- | --- | --- | --- | --- | --- | --- | --- | --- | --- |
|  | | | | | | | | | | | | |
| None | 0 | 1 | 2 | 3 | 4 | 5 | 6 | 7 | 8 | 9 | 10 | Worst  Possible |

Mild	Moderate	Severe	Very Severe

| Depression (being sad or blue) | | | | | | | | | | | | |
| --- | --- | --- | --- | --- | --- | --- | --- | --- | --- | --- | --- | --- |
|  | | | | | | | | | | | | |
| None | 0 | 1 | 2 | 3 | 4 | 5 | 6 | 7 | 8 | 9 | 10 | Worst  Possible |

Mild	Moderate	Severe	Very Severe

| Shortness of breath | | | | | | | | | | | | |
| --- | --- | --- | --- | --- | --- | --- | --- | --- | --- | --- | --- | --- |
|  | | | | | | | | | | | | |
| None | 0 | 1 | 2 | 3 | 4 | 5 | 6 | 7 | 8 | 9 | 10 | Worst  Possible |

Mild	Moderate	Severe	Very Severe

| Neuropathy (numbness, tingling, and/or burning sensation) | | | | | | | | | | | | |
| --- | --- | --- | --- | --- | --- | --- | --- | --- | --- | --- | --- | --- |
|  | | | | | | | | | | | | |
| None | 0 | 1 | 2 | 3 | 4 | 5 | 6 | 7 | 8 | 9 | 10 | Worst  Possible |

Mild	Moderate	Severe	Very Severe

Overall well-being

| Best | 0 | 1 | 2 | 3 | 4 | 5 | 6 | 7 | 8 | 9 | 10 | Worst  Possible |
| --- | --- | --- | --- | --- | --- | --- | --- | --- | --- | --- | --- | --- |

Mild	Moderate	Severe	Very Severe

 **Supplemental Material 4**

**Appendix 1**
